# Implant Treatment After Traumatic Tooth Loss: A Retrospective Cohort Study of Survival, Esthetic, and Patient‐Reported Outcome

**DOI:** 10.1002/cre2.70221

**Published:** 2025-11-18

**Authors:** Frej Nørgaard Petersen, Morten Dahl, Mandana Hosseini, Simon Storgård Jensen

**Affiliations:** ^1^ Department of Oral and Maxillofacial Surgery, Centre of Head and Orthopaedics Copenhagen University Hospital – Rigshospitalet Copenhagen Denmark; ^2^ Oral Rehabilitation, Section for Oral Health, Society and Technology, Department of Odontology, Faculty of Health and Medical Sciences University of Copenhagen Copenhagen Denmark; ^3^ Oral Surgery, Section for Oral Biology and Immunopathology, Department of Odontology, Faculty of Health and Medical Sciences University of Copenhagen Copenhagen Denmark

**Keywords:** Dental implants, maxilla, maxillofacial injuries, tooth loss

## Abstract

**Objective:**

Evidence on biological, technical, and esthetic outcomes following dental implant treatment in the anterior maxilla after traumatic tooth loss is limited. Therefore, this study aimed to evaluate the survival, esthetic, and patient‐reported outcome measures of implant treatment in the anterior maxilla after up to 9 years of functional loading.

**Material and Methods:**

The study was conducted at Copenhagen University Hospital, Denmark. Patients who underwent implant treatment for anterior maxillary tooth loss due to trauma between 2007 and 2019, with at least 1 year of functional loading, were recalled for clinical and radiographic follow‐up.

**Results:**

In total, 56 implants in 49 patients were included. The mean follow‐up period was 4.2 years (range 1–9.5 years). Implant and superstructure survival rates were 100%. Between baseline and the latest follow‐up, there was no statistically significant change in radiographic crestal bone level, but a statistically significant improvement in papilla index. Although not significant, soft tissue texture appeared to improve, while slight soft tissue discoloration was observed in most patients, but remained unchanged from baseline to follow‐up. Crown esthetics generally declined from baseline to follow‐up, although not significantly. Correlation analysis indicated an association between esthetic outcomes and several variables, such as age, gender, number of lost teeth, type of bone defect, and complications before loading. In total, 14% of implants exhibited crown infraposition at follow‐up. No predictive factors for crown infraposition could be identified. Patient‐reported outcome measures generally revealed satisfaction with the treatment results.

**Conclusions:**

The present study found that the biological, technical, and esthetic outcomes of dental implant treatment in the anterior maxilla following traumatic tooth loss are, in general, stable and satisfactory to both clinician and patient. To achieve optimal results in these complex cases, interdisciplinary treatment planning is essential.

## 1 Introduction

Dental implant treatment in the anterior maxilla after traumatic tooth loss is considered a complex, multidisciplinary task. Traumatic dental injuries (TDIs) are most frequently observed in children (8–12 years) (Skaare and Jacobsen 2003; Petti et al. 2018). Although the majority of TDIs have a good prognosis (Andreasen and Ravn 1972), some types (intrusion and avulsion) are at a higher risk of ankylosis‐related and infection‐related resorption (Andreasen et al. 2006a, 2006b, 2006c; Ravn 1974). The pediatric dentist should carefully monitor the patient at appropriate intervals, and often endodontic competencies are required. In many cases, the traumatized tooth may be kept, as long as signs of these irreversible pathological conditions do not develop (Storgård Jensen 2019).

Because treatment with dental implants is contraindicated until completed growth, space and occlusion must often be ensured for several years (Storgård Jensen 2019; Thilander et al. 1994; Chang and Wennström 2012). Therefore, an orthodontist is a crucial member of the team.

Different treatment options exist to replace missing teeth after TDI. Apart from dental implant placement, orthodontic space closure, resin‐bonded bridge, autotransplantation, and tooth‐ or implant‐supported fixed dental prosthesis may be considered (Akhlef et al. 2018; Kiliaridis et al. 2016; Thilander 2008; Bawa et al. 2023; Kern 2017). Oral surgeons should be part of the treatment planning as soon as possible if orthodontic space closure is not an obvious solution. If the idea is to pursue later implant treatment, there should be a clear orthodontic and surgical plan for space and retention (Olsen and Kokich 2010; Thilander 2001). Lastly, a prosthodontist is a mandatory team member when planning any prosthetic tooth replacement and should be consulted in the planning phase as well.

The majority of TDI is inflicted on the anterior maxilla, damaging the teeth and the supporting hard and soft tissues. Therefore, esthetics are a high concern among both professionals and patients (Skaare and Jacobsen 2003; Petti et al. 2018; Glendor 2008). A previous comprehensive review showed a scarcity of reporting clearly defined esthetic parameters (Belser et al. 2004). A more recent review investigated the biological, technical, and esthetic outcomes of implant treatment in the anterior maxilla after traumatic tooth loss (Nørgaard Petersen et al. 2022). Important parameters and outcomes such as pre‐surgical orthodontics, infraposition, patient‐reported outcome measures (PROMs), and complications were only seldom reported. None of the included studies used a validated professional esthetic assessment tool or reported on the use of soft tissue augmentation. A recent consensus paper stated that soft tissue augmentation should be considered in esthetic areas and recommended the use of PROMs as an esthetic outcome measure (Thoma et al. 2021).

Therefore, the aim of the present study was to report the outcome of implant treatment after TDI in the anterior maxilla, with special emphasis on implant survival, esthetic presentation, complications, and patient‐reported outcome up to 9 years after functional loading.

## 2 Materials and Methods

This observational study was prepared according to Preferred Reporting Items for Observational studies in Endodontics (PROBE) 2023 guidelines (Nagendrababu et al. 2023).

The study design was based on a retrospective clinical protocol focusing on quality assessment of the current treatment protocol at the Department of Oral and Maxillofacial Surgery, Copenhagen University Hospital, Denmark. The study was exploratory, and no sample size calculation was performed.

Approval was obtained from the Danish Data Protection Agency (approval number: P‐2020‐612). The revised principles of the Declaration of Helsinki were followed, and written informed consent was obtained from each patient before participation.

Recruitment and data collection took place from December 2020 to June 2021 during a single follow‐up visit at the Department of Oral and Maxillofacial Surgery, Copenhagen University Hospital, Denmark. A standardized recall protocol was followed.

Inclusion criteria: All patients treated by the same surgeon (S.S.J.) with dental implants after traumatic tooth loss in the anterior maxilla, with functional loading of the implant(s) for a minimum of 1 year, were potentially eligible for the study.

Exclusion criteria: Relocation outside the Capital Region, no‐show/cancellation at follow‐up, nonparticipation, nonresponse to recall. Missing baseline data (T0) resulted in exclusion from statistical analysis.

In cases where an implant of the desired dimensions could not be placed in the correct 3D position for the later prosthetic rehabilitation, a staged bone augmentation procedure using an autogenous block graft from the mandible was performed. After 6 months of graft consolidation, implant placement followed using either Straumann Bone Level implants (Straumann AG, Basel, Switzerland) or Astra Tech OsseoSpeed EV implants (Dentsply Sirona, Mölndal, Sweden). The surgical implant procedures were performed according to the manufacturer's recommendations under sterile conditions in an outpatient environment by one of the authors, with more than 20 years of experience in implant dentistry. A conical healing abutment (Straumann SC Healing abutment standard connections or Astra Tech EV Healing abutment) was placed, and the flap was repositioned and sutured to allow a tension‐free healing. In cases with dehiscence‐type defects, a cover screw was mounted, followed by submerged healing. When fenestration‐ or dehiscence‐type defects were present, simultaneous contour augmentation was performed using guided bone regeneration (GBR) by means of locally harvested autogenous bone chips applied on the exposed implant threads and subsequently covered with demineralized bovine bone mineral (DBBM) (Bio‐Oss® Granules 0.25‐1 mm, Geistlich Pharma AG, Wolhusen, Switzerland). Thereafter, the grafted area was covered by a double‐layer collagen membrane (Bio‐Gide, Geistlich Pharma AG, Wolhusen, Switzerland) as described by Buser and colleagues (Buser et al. 2009). In cases of facial bone wall thickness after implant osteotomy of < 1.7 mm, GBR was performed using DBBM alone, covered with a double‐layer collagen membrane on the buccal aspect to protect against additional resorption and to support the soft tissue contour. Details of the surgical procedure have been previously reported (Roccuzzo et al. 2021). After at least 3 months of healing, the implants were clinically and radiographically evaluated by the surgeon. Participants were then referred for prosthetic treatment to the Section of Oral Rehabilitation at the Department of Odontology, Copenhagen University.

All patients were recalled to baseline (T0: Time of loading of the implant with the final superstructure) and at least 1‐year examinations after loading of implants (T1: Follow‐up visit), which included clinical, radiographic, and patient‐reported measurements. Additionally, demographic and clinical baseline characteristics such as patient age at implant insertion time, gender, oral hygiene and care habits, smoking habits, health status at implant placement, number of teeth lost, tooth type lost, trauma mechanism, trauma diagnosis, number of implants, implant type, diameter and length, implant site, mesio‐distal space, presurgical orthodontics, interincisal contact, bone augmentation, type of bone defect at implant placement, and/or bone augmentation (dehiscency, fenestration, thin buccal bone, vertical, or horizontal deficiency), soft tissue augmentation, complications before loading were collected retrospectively through searches in patient medical records and radiographs. Trauma mechanisms were subjectively reported if not recorded.

Follow‐ups included the following examinations

**Figure 1:**
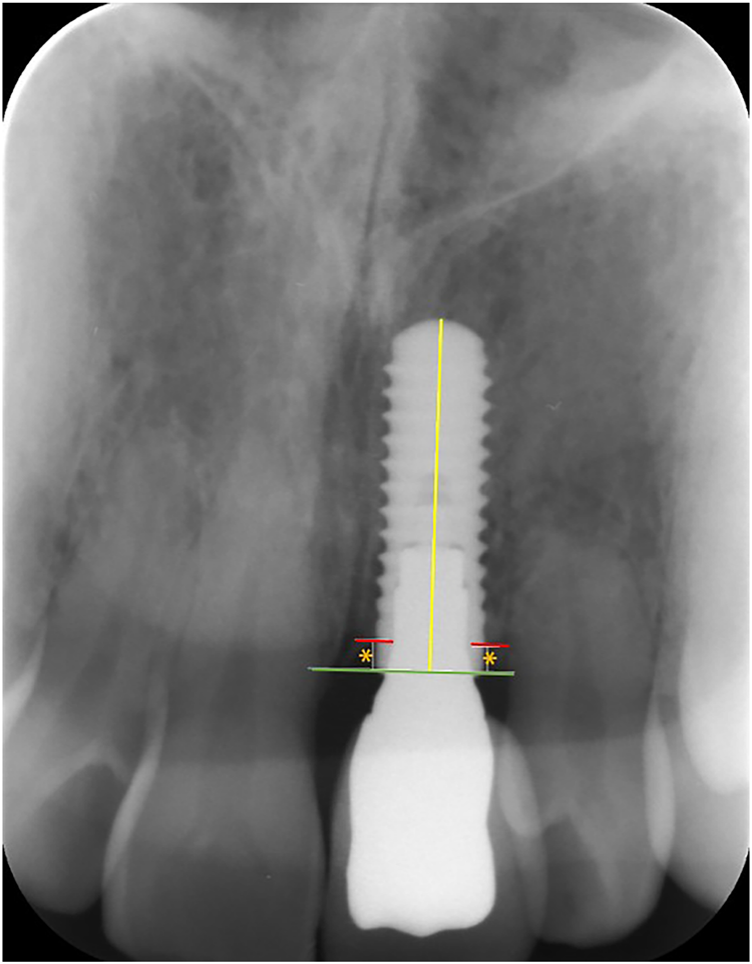
Radiographic marginal bone level measured as linear distance (orange asterisk) from implant shoulder (green line) to first bone‐to‐implant contact (red line). Calibration was done according to the implant length (yellow line).

- Clinical examinations, including registration of implant survival, superstructure survival, peri‐implant tissue health measured by pocket probing depth and bleeding on probing, and rate of complications.
- Clinical photos were taken at baseline and follow‐up according to a standard protocol of four pictures: Smile photo, overview photo in occlusion with cheeks and lips retracted, close‐up photo of the anterior maxillary teeth, and close‐up photo in excentric angle for assessment of atrophy.
- Professional esthetic evaluations were based on the clinical photos using a validated index, the Copenhagen Index Score, and four additional esthetic parameters (Dueled et al. 2009; Hosseini and Gotfredsen 2012).
- Radiographic examination: to assess crestal bone levels and register of any changes over the observation period. Radiographs were taken with intraoral photostimulable phosphor plates (41x31mm) using the paralleling technique. One patient had orthopantomogram done, Planmeca Promax Pan (field of view 30 × 14 cm). Radiographic crestal bone level was measured using the image processing software Image J (Fiji, Wayne Rasband, NIH, US) after calibration according to known implant length (Figure 1).
- Patient‐reported outcome measures: Subjective esthetics were measured using a non‐validated questionnaire at follow‐up visit (T1) (Supporting Information: Appendix A).

One blinded independent assessor (F.N.P.), not involved in the surgical or prosthetic part of the treatment, evaluated all radiographs and clinical photos. Fifteen randomly chosen cases were used for calibration of the esthetic evaluation (Copenhagen Index Score) with an experienced assessor (MH). The esthetic evaluations included 10 esthetic parameters: 6 parameters of the Copenhagen Index Score (CIS) – harmony and symmetry scores, crown morphology score, crown color match score, mucosal discoloration score, and papilla index score (mesially and distally) − 4 additional parameters: level of soft tissue margin, soft tissue curvature, and alveolar process deficiency. These additional esthetic parameters used in this study were based on modified versions of other esthetic scores (Belser et al. 2009; Czochrowska et al. 2002; Fürhauser et al. 2005; Jemt 1997; Juodzbalys and Wang 2010; Meijer et al. 2005; Testori et al. 2005). Each esthetic parameter was categorized using a four‐point rating scale:

Score 1: Excellent

Score 2: Slight mismatch

Score 3: Moderate mismatch

Score 4: Poor esthetic with distinguishable mismatch compared to regions with natural healthy teeth.

Symmetry/harmony was assessed according to the facial midline, the tooth axis, the contralateral tooth, and the smile line. The papilla index score was as follows:

Score 1: Papilla filling the entire proximal space

Score 2: Papilla filling at least half the proximal space

Score 3: Papilla filling less than half the proximal space

Score 4: No papilla

In addition to the four scales, sub‐scores were used for the following parameters: crown morphology: smaller/bigger, crown color match: darker/brighter, mucosal discoloration: gray/red/white/visible abutment, level of soft tissue margin: apical/incisal, soft tissue texture: smoother/rougher, soft tissue curvature: elongated/flattened, alveolar process deficiency: over‐contoured/under‐contoured.

Descriptive and inferential statistics were applied. The ratio of implants per patient was low (1.1:1); therefore, the implant was used as the statistical unit. Potential confounders for the statistical analyses were oral hygiene and care habits, smoking habits, and health status at implant placement. For categorial data, frequencies (absolute and relative) were calculated, and for continuous data, mean, standard deviation, range, median, and interquartile range were calculated. A Kolmogorov–Smirnov's test was used to test for normal distribution. Wilcoxon's test was used to analyze changes in radiographic bone level and esthetic indices between T0 and T1. McNemar's test was used for binary variables. Binomial and Chi^2^ test was used to analyze relative distributions for subcategories in esthetic indices. Mann–Whitney and Kruskal–Wallis tests were used to compare ordinal variables. Spearman's correlation coefficient was calculated to measure associations between ordinal and/or continuous variables (independent variables and esthetic outcomes). Level of significance was set at 5%. Due to the exploratory nature of this study, no Bonferroni correction was applied.

## 3 Results

**Figure 2:**
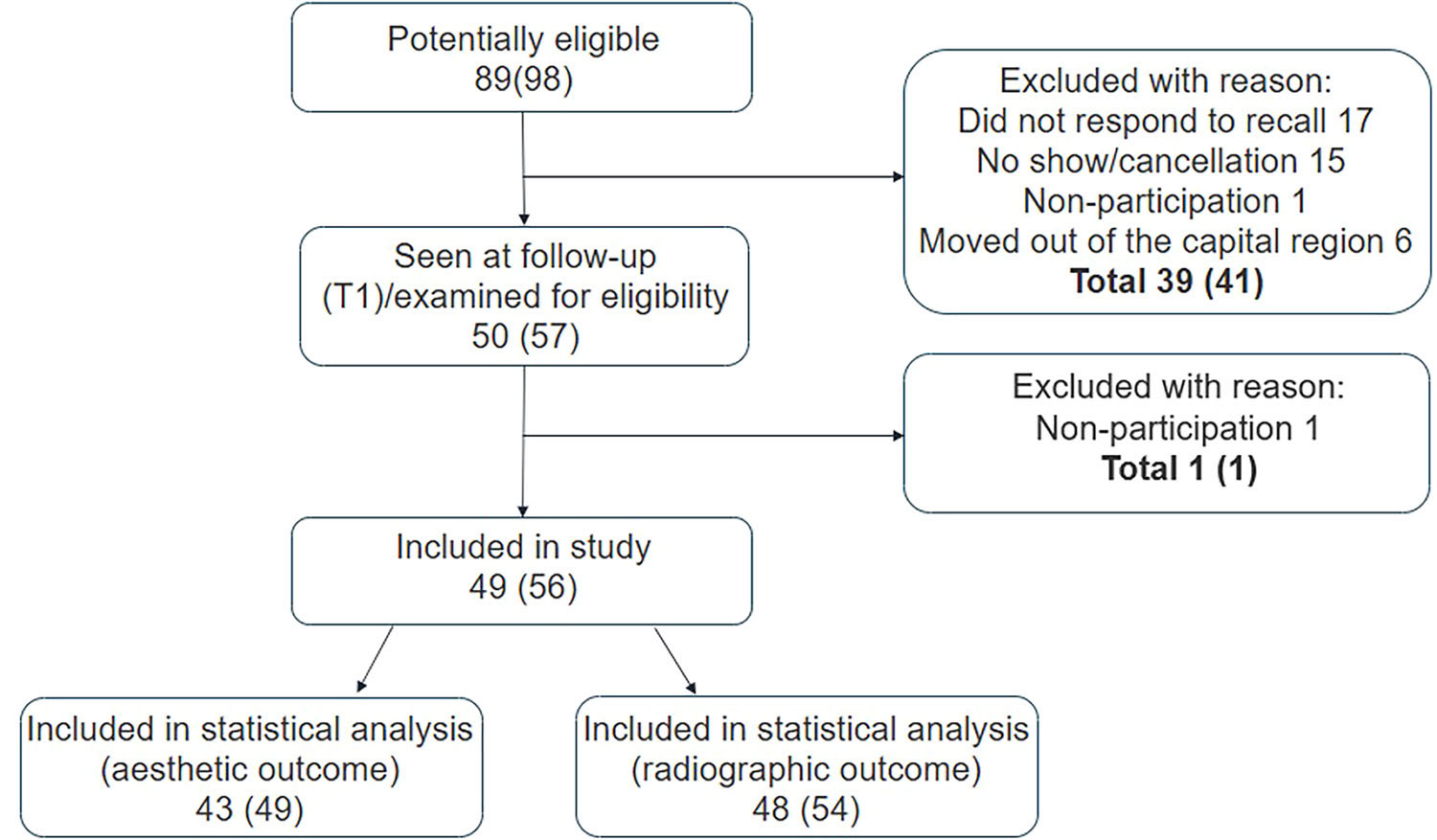
Flow chart of patient inclusion. Number of patients (number of implants).

Patient recruitment and selection were performed according to the flow chart (Figure 2). In total, 49 patients with 56 implants were included. Data from the baseline examination were missing in six patients (seven implants) in the form of photos, and in one patient (two implants) in the form of radiographs, resulting in statistical analysis (comparing T0 and T1) on 43 patients (49 implants) and 48 patients (54 implants) on esthetic outcome and crestal bone loss, respectively.

**Figure 3:**
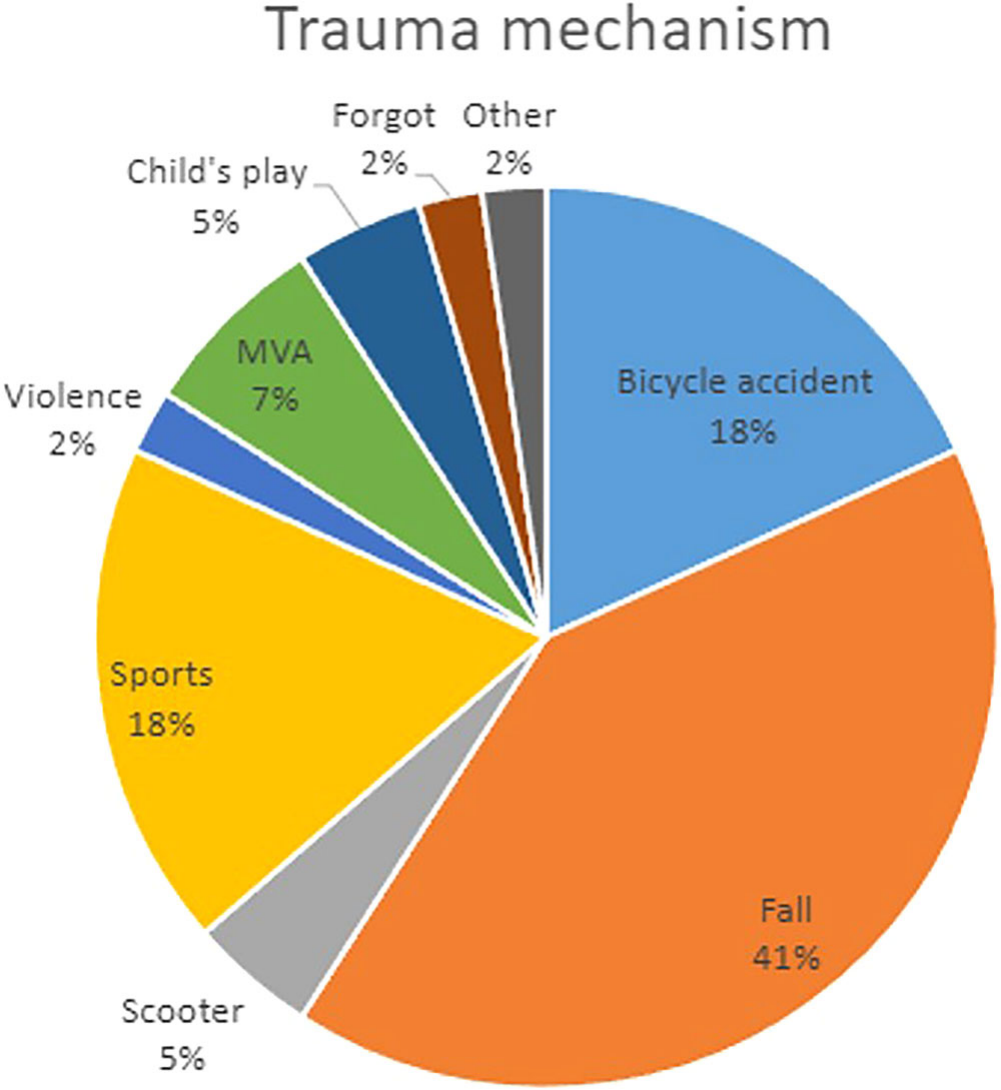
Distribution of trauma mechanism. Trauma mechanism in percentage as reported by patients at follow‐up. MVA = motor vehicle accident.

**Figure 4:**
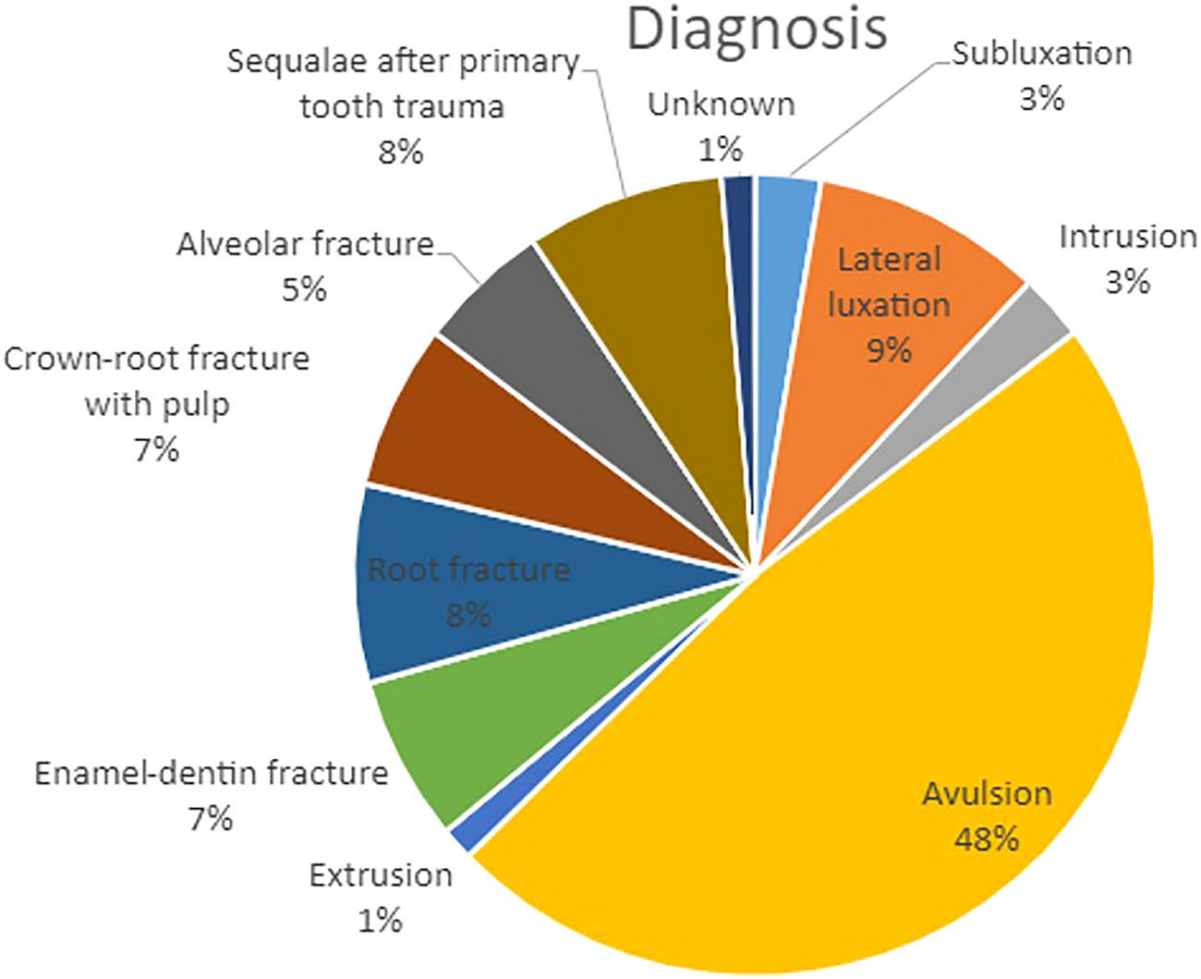
Distribution of tooth trauma diagnosis. Trauma diagnosis in percentage on the tooth level of lost teeth. One or more diagnosis is possible for a single tooth, i.e., luxation and alveolar fracture.

All participants were generally healthy and on average were 22 years old (SD ± 4.1 years). The mean follow‐up was 4.2 years (median 3.7 years, range 1–9.5 years). Ten patients were smokers, of whom four were social smokers (less than one cigarette per day on average). Regarding oral hygiene habits, 80% stated that they attended regular dental check‐ups, 74% brushed twice daily, and 45% performed regular interproximal cleaning. Table 1 summarizes patient and implant characteristics. Distribution of trauma mechanism and diagnosis is shown in Figures 3 and 4, respectively. At baseline, six patients (12.2%) had experienced complications before loading of the implants.

**Table 1:** Patient and implant characteristics, mean (SD)/number (%).

Number of patients/implants	49/56
Age at implant placement	22.0 ± 4.1 years
	(range 18.6–44.2 years)
Sex (M/F)	29M/20F (59%/41%)
Number of lost teeth	63
Tooth type	52 (82.5%) MCI
	10 (15.9%) MLI
	1 (1.6%) MC
Implant type	37 (66%) Straumann
	19 (34%) Astra Tech
Implant length	8 (10 mm), 11 (11 mm)
	29 (12 mm), 8 (13 mm)
Diameter	1 (3.0 mm), 5 (3.3 mm)
	2 (3.6 mm), 32 (4.1 mm)
	14 (4.2 mm), 2 (4.8 mm)
Preoperative orthodontics, yes/no (patient level)	25/24 (51%/49%)
Preoperative interincisal contact, yes/no/NR (patient level)	25/18/6 (51/37/12%)
Preoperative mesio‐distal space	9.02 ± 2.38 mm
Number of bone augmentations (patient level/implant level)	45/52 (92%/93%)
Number of soft tissue augmentations (patient level/implant level)	6/6 (12%/12%)

Abbreviations: F, female; M, male; MC, maxillary canine; MCI, maxillary central incisor; MLI, maxillary lateral incisor; NR, not reported.

All 56 implants and superstructures were still in situ at follow‐up (100% survival rate). Pocket probing depth (PPD) was below 4 mm in 91.9% (mean) of patients at baseline and in 84.7% (mean) of patients at follow‐up. Mesial (*p* = 0.527) and distal (*p* = 0.863) changes were not significant. Bleeding on probing (BOP) was present in 18.2% (mean) at baseline and in 31.6% (mean) at follow‐up. Mesial (*p* = 0.146) and distal (*p* = 0.227) changes were not significant.

**Figure 5:**
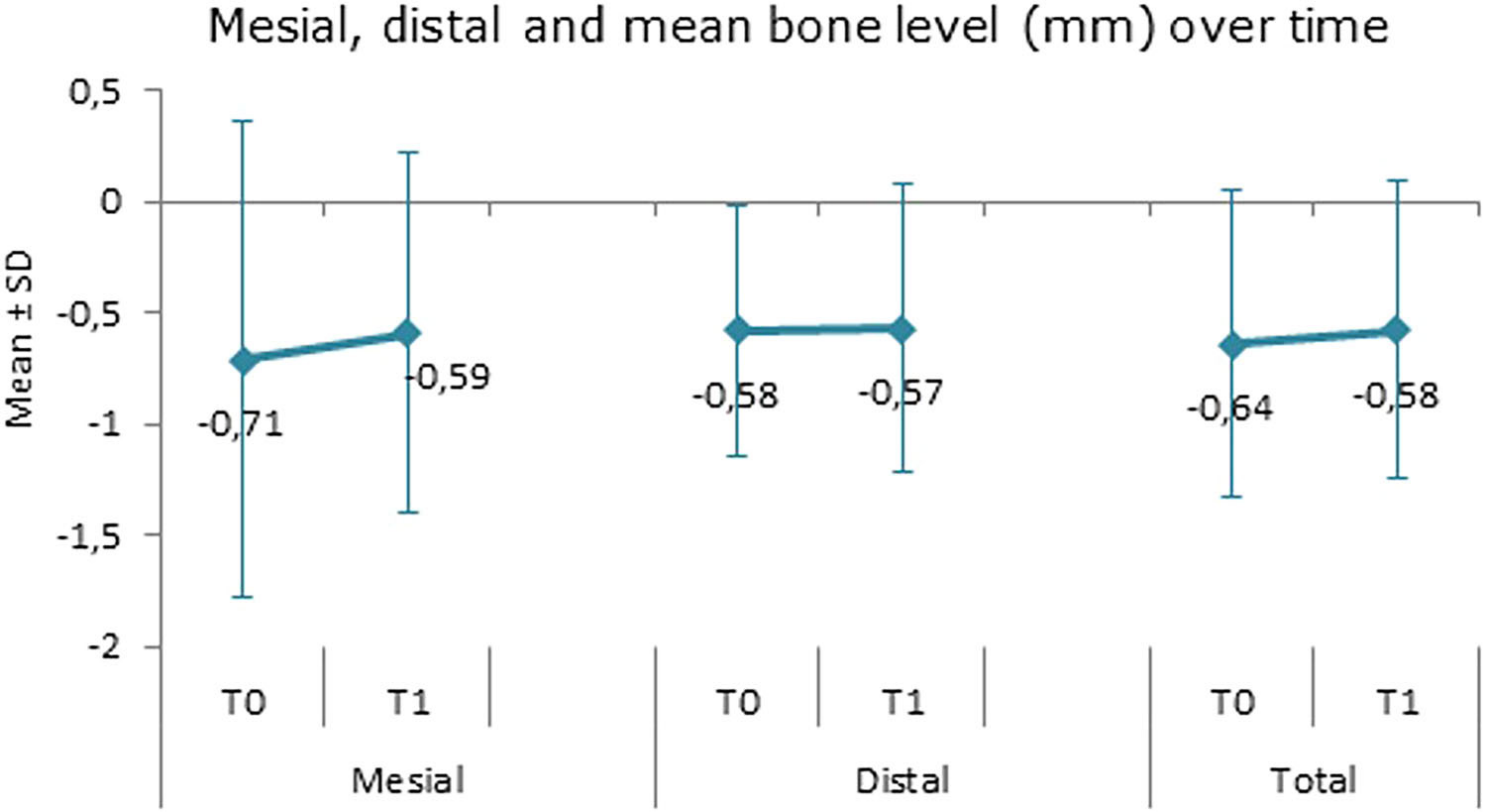
Radiographic marginal bone level change. Mean (SD: standard deviation) radiographic marginal bone level change in mm from baseline (T0) to follow‐up (T1).

Mean radiographic crestal bone change was +0.12 mm (SD ± 0.73 mm, range –1.72 to +2.99 mm, *p* = 0.557) at the mesial aspect and +0.01 mm (SD ± 0.52 mm, range –1.32 to +1.06 mm, *p* = 0.549) at the distal aspect of the implants and thus not statistically significant. The combined mesial and distal changes in crestal bone levels were 0.07 mm (SD ± 0.50 mm, range –1.38 to 1.93 mm, *p* = 0.296) (Figure 5). Estimated crestal bone level changes per implant per year were +0.029 and +0.002 mm at the mesial and distal aspects, respectively. Two outliers at baseline showed a negative marginal bone level of 5 and 7 mm, respectively. At follow‐up, no outliers were observed. Lowest measure at follow‐up was a marginal bone level of −4 mm at two implants.

The esthetic evaluation showed excellent to satisfactory (symmetry/harmony scores 1 and 2) results in 95.9% and 91.8% of the implant cases at T0 and T1, respectively.

Symmetry/harmony score 3 increased from T0 to T1 by approximately 4%, although not significant (*p* = 0.475).

Papilla index scores (mesial and distal) 3 and 4 (i.e., fill less than half of proximal space and completely missing papilla) were seen in approximately 14%–16% and 2%–4% of cases at T0, respectively. At T1, scores 3 and 4 decreased significantly to approximately 6%–10% and 0%–4%, respectively (mesial, *p* = 0.009; distal, *p* = 0.015).

Soft tissue texture score 3 (i.e., moderate mismatch) increased from T0 to T1, although not significant (*p* = 0.178). Sub‐scores showed significantly more cases were assessed as “rougher” than “smooth” at both T0 (*p* < 0.001) and T1 (*p* < 0.001), but the total number of suboptimal cases decreased by 10 from T0 to T1. Details are provided in Table 2.

**Table 2:** Esthetic and radiographic evaluation on the implant level. Frequency of scores at baseline (T0) and follow‐up (T1).

Esthetic parameters						
	Score	T0 (*n* = 49)	T1 (*n* = 49)	T0–T1 Change in scores		*p*‐value
Symmetry/harmony	1	59.2%	57.1%	−1	12.2%	0.475
	2	36.7%	34.7%	0	73.5%	
	3	4.1%	8.2%	1	10.2%	
	4	0%	0%	2	4.1%	
Crown morphology	1	59.2%	44.9%	−1	6.1%	0.052
	2	40.8%	55.1%	0	73.5%	
	3	0%	0%	1	20.4%	
	4	0%	0%			
Crown morphology sub‐score		*n* = 20	*n* = 27			
Smaller	1	25%	48.1%			
Bigger	2	75%	51.9%			
Crown color match	1	59.2%	49.0%	−1	4.1%	0.058
	2	40.8%	49.0%	0	79.6%	
	3	0%	2.0%	1	16.3%	
	4	0%	0%			
Crown color match sub‐score		*n* = 20	*n* = 25			
Darker	1	90%	68%			
Brighter	2	10%	32%			
Mucosal discoloration	1	14.3%	12.2%	−1	4.1%	0.655
	2	85.7%	87.8%	0	89.8%	
	3	0%	0%	1	6.1%	
	4	0%	0%			
Mucosal discoloration sub‐score		*n* = 42	*n* = 43			
Gray	1	59.5%	69.8%			
Red	2	16.7%	20.9%			
White	3	23.8%	9.3%			
Visible abutment	4	0%	0%			
Papilla index, mesial	1	42.9%	61.2%	−2	2.0%	0.009*
	2	38.8%	28.6%	−1	32.7%	
	3	14.3%	6.1%	0	55.1%	
	4	4.1%	4.1%	1	10.2%	
				
Papilla index, distal	1	22.4%	36.7%	−2	2.0%	0.015*
	2	59.2%	53.1%	−1	28.6%	
	3	16.3%	10.2%	0	63.3%	
	4	2.0%	0%	1	4.1%	
				2	2.0%	
Level of facial mucosal margin	1	53.1%	53.1%	−1	14.3%	0.782
	2	42.9%	44.9%	0	73.5%	
	3	4.1%	2%	1	12.2%	
	4	0%	0%			
Level of facial mucosal margin sub‐score		*n* = 23	*n* = 23			
Apical	1	52.2%	60.9%			
Incisal	2	47.8%	39.1%			
Soft tissue texture	1	38.8%	59.2%	−2	2.0%	0.178
	2	59.2%	32.7%	−1	28.6%	
	3	2.0%	8.2%	0	51.0%	
	4	0%	0%	1	18.4%	
Soft tissue texture sub‐score		*n* = 30	*n* = 20			
Smoother	1	6.7%	5.0%			
Rougher	2	93.3%	95.0%			
Soft tissue curvature	1	67.3%	65.3%	−1	12.2%	0.782
	2	32.7%	34.7%	0	73.5%	
	3	0%	0%	1	14.3%	
	4	0%	0%			
Soft tissue curvature sub‐score		*n* = 16	*n* = 17			
Elongated	1	18.8%	23.5%			
Flattened	2	81.3%	76.5%			
Alveolar process deficiency	1	44.9%	38.8%	−1	12.2%	0.405
	2	53.1%	55.1%	0	65.3%	
	3	2.0%	6.1%	1	22.4%	
	4	0%	0%			
Alveolar process deficiency sub‐score		*n* = 27	*n* = 30			
Overcontoured	1	14.8%	16.7%			
Undercontoured	2	85.2%	83.3%			
**Radiographic parameters**						
Cement excess		*n* = 54	*n* = 54	−1	1.9%	1.000
	0	98.1%	98.1%	0	96.3%	
	1	1.9%	1.9%	1	1.9%	
Marginal adaptation		*n* = 54	*n* = 54	−1	0.0%	0.014*
	1	92.6%	83.0%	0	88.9%	
	2	7.4%	14.8%	1	11.1%	
	3	0%	1.9%			
	4	0%	0%			

*Note:* Category descriptions published elsewhere (Dueled et al. 2009; Hosseini and Gotfredsen 2012). Main scores were compared between timepoints T0 and T1. Sub‐scores were compared within timepoints T0 and T1, respectively. Statistically significant *p*‐values are marked with *p < 0.05.

The correlation analysis showed a significant relationship between several variables (age, gender, number of lost teeth, tooth type, number of implants, mesio‐distal space, pre‐surgical orthodontics, interincisal contact, implant type, diameter and length, bone augmentation (yes/no and timing), type of bone deficiency, complications before loading, length of follow‐up) and esthetic outcomes and radiographic outcomes (cement excess and marginal adaption).

Younger patients scored significantly better in crown morphology at T0 (*p* = 0.033), but not at T1 (*p* = 0.234), and had significantly darker crowns more often in cases with suboptimal crown color match than older patients at T0 (*p* = 0.019) and T1 (*p* = 0.004). Gender was associated with a significantly different distribution of change in symmetry/harmony. Females improved in 27.3% of cases compared to 0% in males from T0 to T1 (*p* = 0.016). Soft tissue color was better in females at T0 and T1 (*p* = 0.023, *p* = 0.005, respectively). The number of lost teeth was significantly associated with esthetic scores. Single tooth loss, compared to multiple tooth loss, was associated with better scores in symmetry/harmony, crown morphology, soft tissue curvature, and papilla index at T0 (*p* = 0.030) but not at T1 (*p* = 0.721).

The number of implants was significantly associated with esthetic scores. Crown morphology score was better at T0 for single tooth loss (*p* = 0.048). Soft tissue curvature and papilla index scores (mesially) were better at T0 and T1 (*p* < 0.001 and *p* < 0.001, *p* = 0.001 and *p* < 0.001, respectively). Papilla index (distally) was better at T0 (*p* = 0.030) but not at T1 (*p* = 0.281). The implant diameter was significantly associated with improvement in soft tissue color from T0 to T1 (*p* = 0.018). Complications before loading (wound dehiscence (*n* = 2), infections (*n* = 2), fistula (*n* = 1), and resorption of bone augmentation (*n* = 1)) negatively affected symmetry/harmony, crown morphology, papilla index, and soft tissue curvature. All scored better in the absence of complications at both T0 and T1. Alveolar process deficiency scored better in implants followed for a shorter time (≤ 3 years) at T1 (*p* < 0.025).

**Figure 6:**
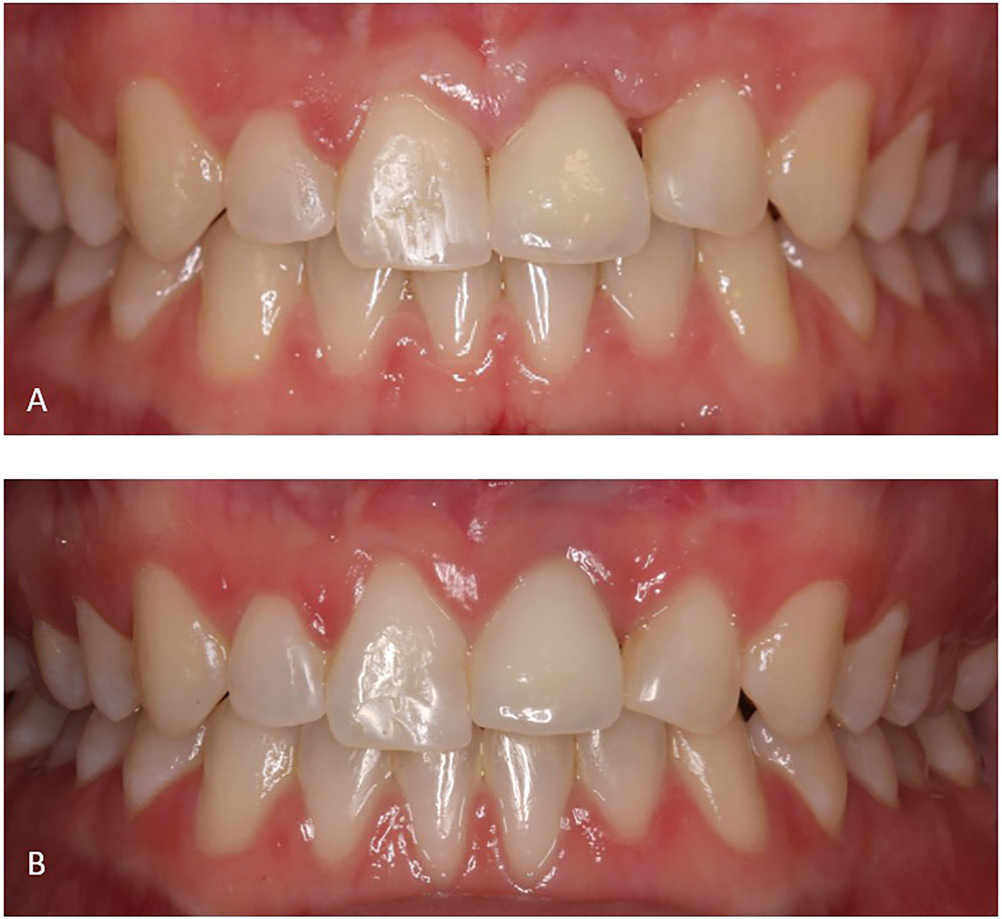
Example of infraposition and esthetic outcome. Photos of implant crown left maxillary central incisor at (A) baseline (T0) and (B) 4 years follow‐up, note the increase in infraposition of the implant crown and the red mucosal discoloration as a result of gingival inflammation and insufficient oral hygiene (T1).

At follow‐up, 18 patients experienced a total of 22 complications, which were calculated at the implant level (39.4%). The complications were esthetic, biological, and prosthetic (Table 3). Infraposition was recorded on eight implants. Regarding the risk of infraposition in cases of missing interincisal contact, no correlation was found (*p* = 0.120); in fact, infraposition was reported more often in cases with interincisal contact (*n* = 4). One patient without interincisal contact at baseline suffered the highest degree of infraposition recorded of about 1.5 mm after 4 years of loading (Figure 6). The patient was female and 20.5 years old at implant placement. Additionally, the photo at follow‐up shows marginal discoloration and improved papilla fill mesially and distally.

**Table 3:** Esthetic, biologic, and prosthetic complications at follow‐up (T1).

Complication		
Esthetic	Infraposition	8 (14.3%)
	Implant crown form and/or color not a fit	1 (1.8%)
Biological	Atrophy of peri‐implant hard and/or soft tissue	6 (10.7%)
	Peri‐implantitis	1 (1.8%)
	Fistula without pus	2 (3.6%)
Prosthetic	Ceramic chipping	2 (3.6%)
	Abutment loosening	1 (1.8%)
	Implant crown not seated properly	1 (1.8%)
Total		22 (39.4%)

*Note:* Complications at the implant level. Three of the patients who presented with complications had two implants each. The respective complications concerned only one implant in each patient. Therefore, number of complications is the same at1 patient level.

**Figure 7:**
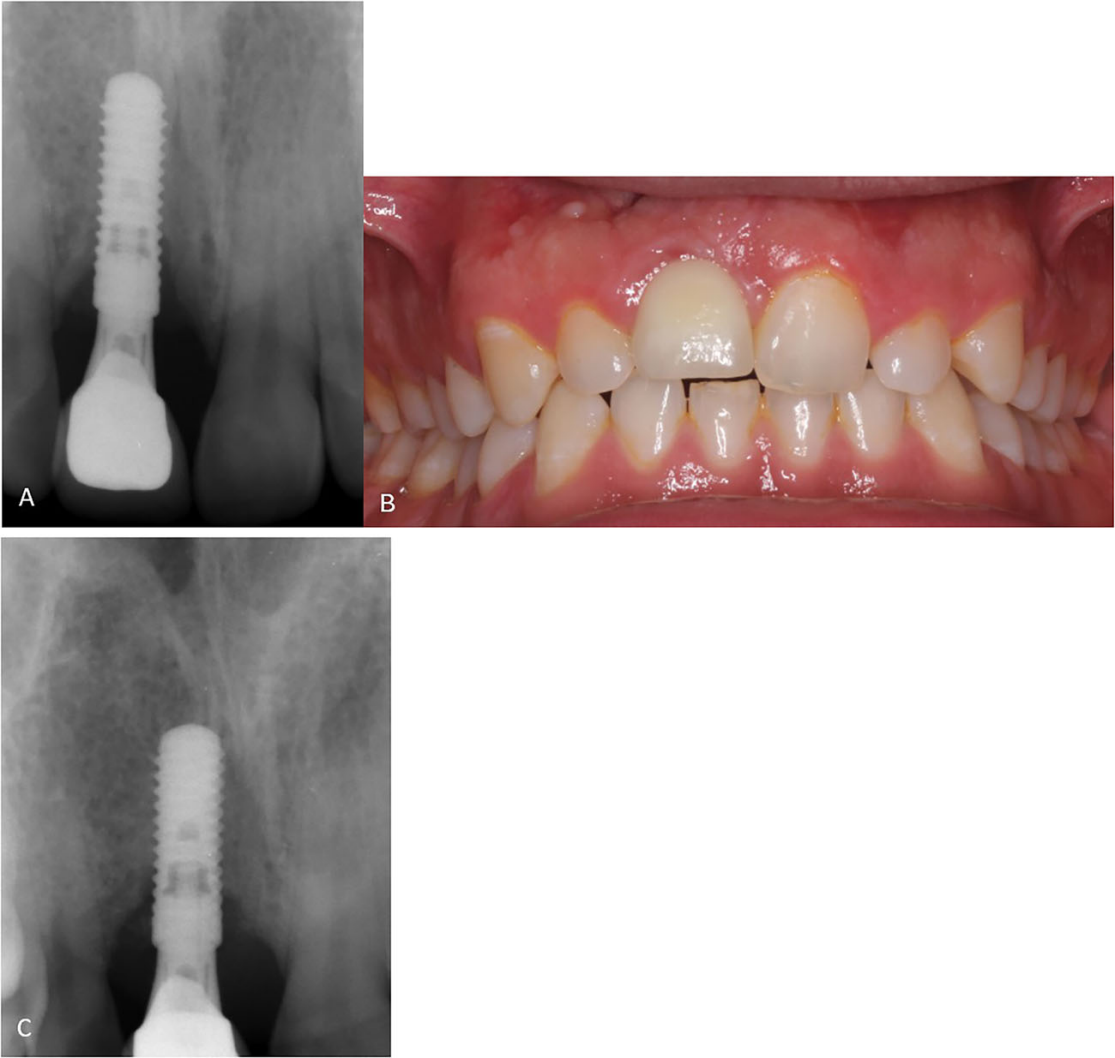
Example of radiographic marginal bone loss. This patient experienced a complication of peri‐implantitis on the implant in the right maxillary central incisor region approximately 4 years after loading (T1). (A) Before peri‐implantitis treatment at follow‐up (T1), (B) Photo at T1, note fistula at muco‐gingival margin, red mucosal discoloration, and full papilla fill, (C) 2 years after peri‐implantitis treatment, note healing of mesial bone defect.

One patient suffered peri‐implantitis approximately 4 years after loading. The patient was a healthy male and a former smoker. Radiographically, a vertical defect around one‐third of the implant length at the mesial aspect could be noted (Figure 7A). At implant placement, a dehiscence‐type defect was noted and simultaneously augmented according to the surgical protocol. At abutment surgery, a small facial wound dehiscence was noted. The following months, a noninfectious fistula, which exfoliated xenograft particles was observed. Spontaneous healing occurred and the superstructure was mounted. After 4 years, the patient received operative peri‐implantitis treatment (Figure 7B). After 2 years, radiographic improvement was seen (Figure 7C).

**Figure 8:**
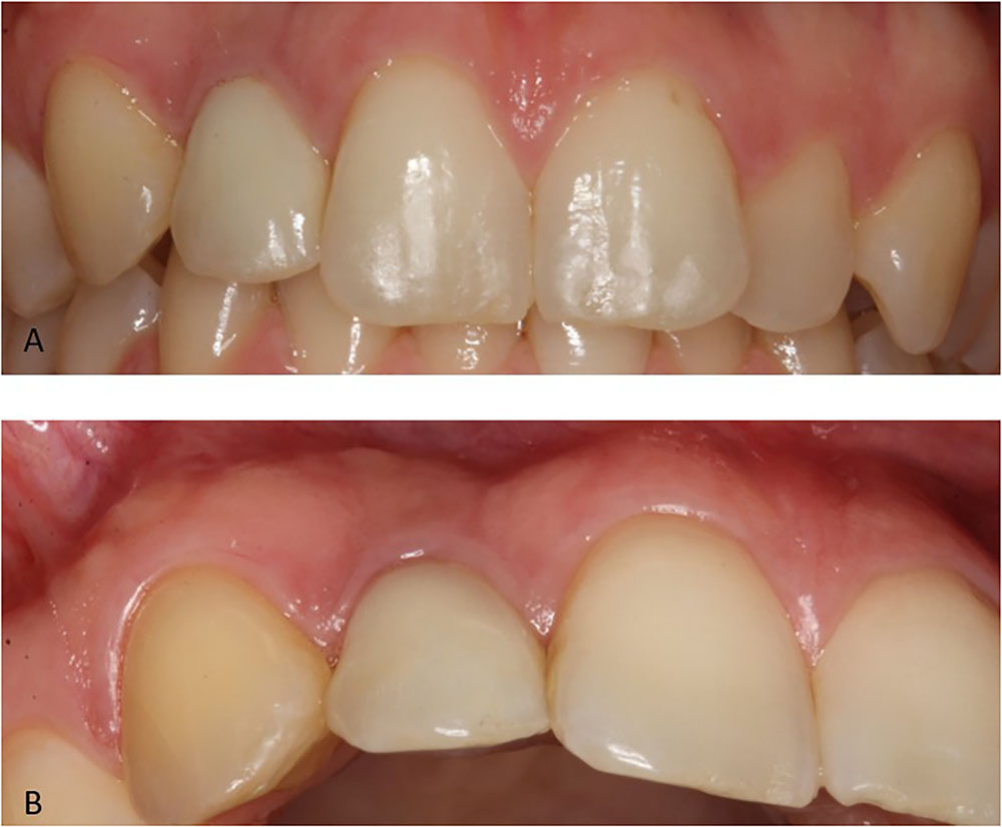
Example of atrophy. Implant in the right maxillary lateral incisor region at follow‐up, showing dark mucosal discoloration and atrophy of the hard and soft tissues. (A) Close‐up view and (B) eccentric view.

At the 5‐year follow‐up, one patient (female, age at implant placement: 18.9 years) was not completely satisfied with the esthetics due to shine‐through, causing dark discoloration of the mucosa. The clinical examination demonstrated atrophy of the alveolar process at the buccal aspect of the implant, although a particulate bone augmentation was performed simultaneously with implant placement (Figure 8). The patient was offered a soft tissue augmentation procedure.

**Figure 9:**
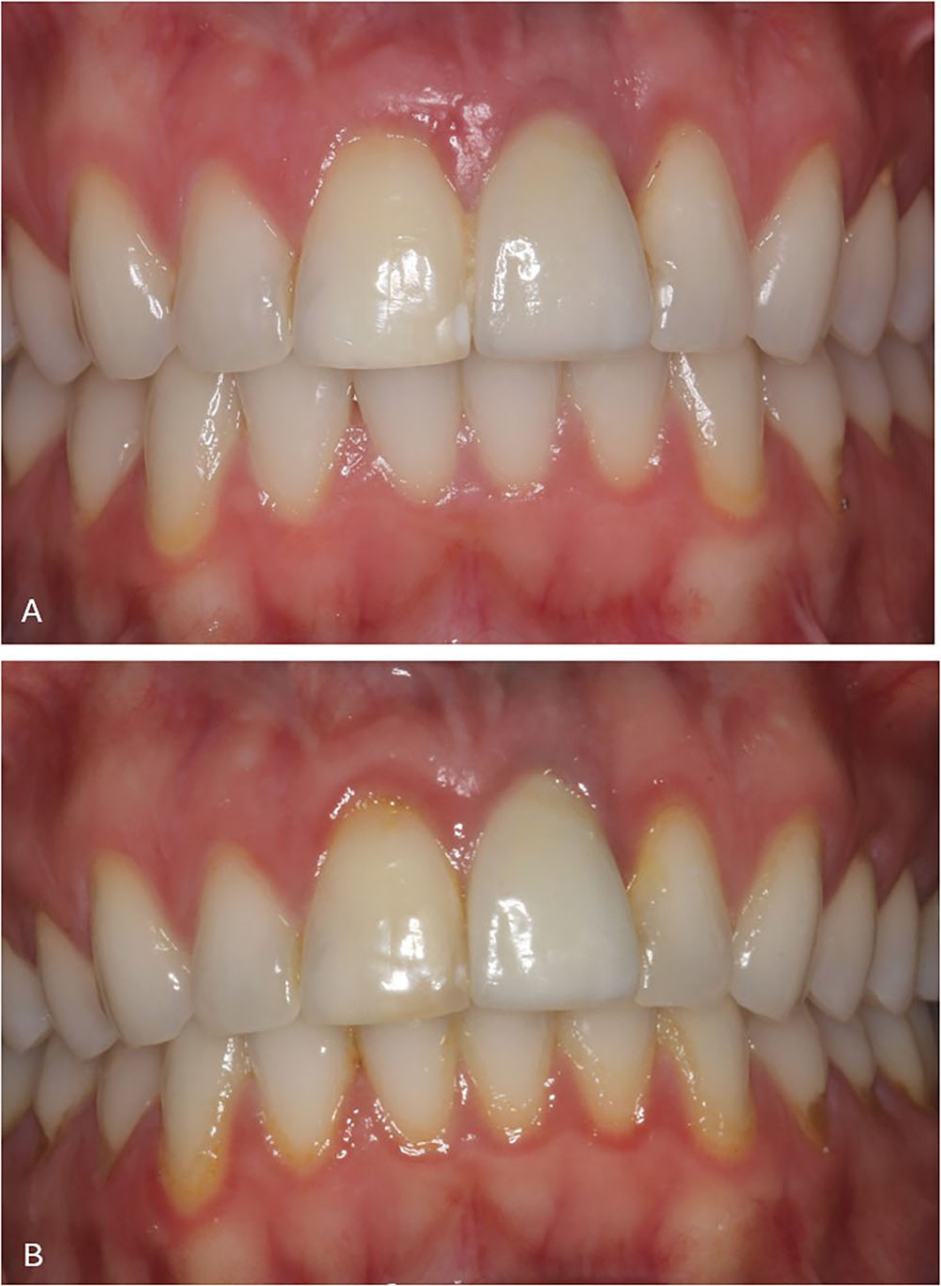
Example of patient‐reported outcome measures. Implant in the left maxillary central incisor region showing the patient's esthetic complaints regarding crown color (too white), mucosal discoloration, atrophy of the peri‐implant mucosa, and incomplete distal papilla fill. (A) Baseline (T0) and (B) 3.5 years follow‐up (T1).

One of the patients (female) received a single implant in the central incisor region. She had presurgical orthodontics, interincisal contact, 8 mm mesio‐distal space, and independent bone augmentation with block graft because of vertical and horizontal deficiency. At 3.5 years follow‐up, she responded dissatisfaction with the crown color and form, implant position in the tooth arch, mucosa color (too dark), gingival volume (too flat), and papilla fill (too little) (Figure 9). She was offered a new superstructure and a soft tissue augmentation.

Regarding PROMs, 83% and 73% of patients were satisfied with the esthetics of the implant crown and the soft tissue, respectively. 41% of patients answered that the implant looked similar to their own teeth, whereas 55% sometimes noticed a difference. 84% of patients never felt limited because of the esthetics of the implant and/or crown. 90% of patients responded that they never avoided smiling because of the esthetics of the implant and/or crown. 75% of patients responded with a score of 9 or 10 on a numeric rating scale (NRS) of 0–10 regarding satisfaction with their treatment. Median NRS was 9. There were a few dissatisfied patients, and their complaints concerned the peri‐implant soft tissue – specifically, the color (too dark), the papilla fill (too little fill), and the volume (too flat).

## 4 Discussion

The present study confirms that implant treatment replacing teeth lost after TDI is a predictable treatment option, characterized by a high survival rate, a low complication rate, and acceptable esthetics from both a professional and a patient‐reported point of view. However, subjective assessment at T1 revealed that 12 patients (32.4%) were not completely satisfied with the crown length, with nine patients (24.3%) reporting that the crown had become too short. This is in line with a recent study, which found that even 0.25–0.5 mm of infraposition of the anterior teeth may negatively affect esthetics, both in the eyes of the professional and the patient (Ntovas et al. 2024). In case of a high smile line (gummy smile), the clinical challenge is then, that changing the superstructure to correct the infraposition will not improve esthetics. The authors suggest that soft tissue augmentation and orthodontics might be necessary as well (Ntovas et al. 2024). The drop in crown color match score and increase in perception of a brighter crown at T1 may be explained by the neighboring teeth becoming opaque over time, because of secondary or tertiary dentine deposition, giving the impression of a brighter implant crown. Interestingly, looking at the sub‐group of younger patients (age at implant insertion ≤ 20 years) with suboptimal scores, most perceived their crown as darker. This study's hypothesis is that, to most patients, a too bright crown might be more acceptable than a too dark crown. Soft tissue color scores did not improve between T0 and T1. It makes sense that the majority of the discolored cases were assessed as gray. Shine‐through of the implant or abutment material is a well‐known esthetic complication in cases of thin buccal bone and/or soft tissue (Jung et al. 2007). This is usually perceived as gray or dark discoloration of the mucosa. Red discoloration was the second most frequent sub‐score and remained relatively constant at T0 and T1 and represents inflammation of the mucosa (peri‐mucositis). Examples of red discoloration due to insufficient oral hygiene can be seen in Figures 6 and 9. Both patients showed generalized plaque accumulation and inflammation at T1, which might have affected the outcome. White discoloration decreased from T0 to T1 and may be explained by maturation of scar tissue (Sculean et al. 2014). This was also indicated by the improvement in the soft tissue texture from T0 to T1, although without reaching statistical significance. There were no cases of visible abutments, which indicates that all implants were placed in an acceptable 3D‐position in the alveolar process, and that the supracrestal height was respected (Buser et al. 2004).

Papilla index scores increased from T0 to T1. Studies indicate that complete papilla fill can be expected in cases with ≤ 5 mm between interproximal bone level and implant crown contact point (Nisapakultorn et al. 2010; Cosyn et al. 2012; Tarnow et al. 2000; Choquet et al. 2001). The interproximal bone level is determined by the inter‐implant or implant‐tooth distance and may also influence papilla fill (Nisapakultorn et al. 2010; Tarnow et al. 2000). Mesial and distal papilla showed complete fill or at least half fill of the proximal space in 89.8% of cases at T1.

The potential biological reaction of poor marginal adaptation and cement excess is inflammation of the peri‐implant tissues and subsequent crestal bone loss (Canullo et al. 2016). Although marginal adaptation worsened from T0 to T1, clinically only one loose abutment was identified. The decrease might then be explained by a difference in radiographic projection between T0 and T1.

Implant and superstructure survival was high and comparable to studies on implant placement in general (Jung et al. 2012; Pjetursson et al. 2014) and after TDI (Nørgaard Petersen et al. 2022). A mean gain in crestal bone level was observed, although clinically negligible and not significant. Between 82% and 84% of the implants had a crestal bone level between –1 and +1 mm from the implant shoulder at T1, which is considered satisfactory (Albrektsson et al. 2017).

A high rate (41.1%) of complications at follow‐up was seen. This is comparable to a study, which found a complication rate of 45% for implant placement after TDI (Schwartz‐Arad and Levin 2004). The high rate might be explained by the wide definition of complications. Complications had no impact on implant or superstructure survival. Infra‐position and atrophy accounted for 60.9% of complications, which impacted the esthetic outcome. To minimize the risk of infraposition, at the authors' department, it is recommended that dental implants are not inserted until arrested skeletal growth has been documented (by two body height measurements at least 1 year apart) and interincisal contact has been ensured (Storgård Jensen 2019). Interestingly, our study found no correlation between infraposition and interincisal contact. This supports the finding in other studies that adults continue to experience growth of the jaws, and teeth adapt relative to the implant (Thilander et al. 1994; Chang and Wennström 2012; Oesterle and Cronin RJ 2000). Because of this, clinicians should inform patients of the risk of compromised esthetics and potential future need for superstructure replacement. Even so, patients might insist on ending the recommended pre‐surgical orthodontic treatment before ideal interincisal contact is achieved or completely refuse orthodontic treatment. In these cases, it must be assessed whether implant treatment is feasible or not, and, if treatment is performed, to inform the patient about a potential increased risk of infraposition. Treatment of severe infraposition is challenging and may range from remaking of the implant‐supported FDP over surgical segmental osteotomy, distraction osteogenesis, or removal of the implant followed by augmentation of hard and/or soft tissue before a new implant can be placed (Zitzmann et al. 2015). Complications before loading were correlated with lower esthetic scores. It could not be determined which type of complication led to a specific esthetic problem, but wound dehiscence may explain the lower scores in soft tissue curvature, and infections or inflammatory processes like peri‐implantitis may explain the lower scores in the papilla index. Young age and female gender seemed to positively influence certain esthetic outcomes. Loss of more than one tooth seemed to be correlated with less favorable esthetic outcomes. This is also the conclusion in the literature (Belser et al. 2004). Implants with simultaneous contour augmentation of dehiscence‐type bone defects seemed to perform better overall, both at T0 and T1, compared to other defects. Not surprisingly, longer follow‐up had a negative impact on the alveolar process deficiency score.

PROMs revealed that the patients expressed overall satisfaction with the esthetic results. This is in line with similar clinical studies (Gotfredsen 2012; Andersson et al. 2003; Vilhjálmsson et al. 2011). A closer look at the responses of less satisfied patients disclosed high levels of expectations regarding the esthetics of the peri‐implant soft tissues. Aspiring to meet patient wishes is a main goal of treatment; therefore, informing patients about individual compromising factors to further align realistic expectations is crucial for patient satisfaction.

Not surprisingly, the reason for tooth loss was avulsion in almost 50% of the teeth (Figure 4), as the prognosis is worse for these teeth (Ravn 1974). Interestingly, in 8% of the teeth, the reason for permanent tooth loss was prior primary tooth trauma. A recent retrospective study of avulsed primary teeth (*n* = 266) in children showed sequelae (any kind, i.e., malformation or root divergence) in 43.2% of the permanent dentition (Del Negro et al. 2022). This underlines the need for early diagnosis and long‐term follow‐up of patients suffering TDI. This study has certain strengths. To the knowledge of the authors, it is the first study to provide systematic evidence regarding the esthetic outcomes of implant treatment after traumatic tooth loss using a validated index. Furthermore, it contributes a relatively large and homogenous cohort of patients, matching the only other relevant publication (Schwartz‐Arad and Levin 2004) reporting on this number of patients to date. To reduce assessor bias, blinding of the esthetic evaluation and radiographic measurements was done. The surgical procedures were performed by the same surgeon and according to a standardized protocol.

The study also has several limitations. Because of the retrospective nature of this study, certain biases should be expected, e.g., selection bias and recall bias. Patients were not randomly selected but included from a single surgeon's database. Trauma mechanism was mostly based on patients' recollection. Confounders and effect modifiers could not be adjusted for and may have affected the outcomes. Additionally, unidentified confounders and modifiers of potential influence are socioeconomic status, patient compliance, bone quality and density, and gingival phenotype. Clinical photos were taken according to the authors' own standardized protocol, but changing light conditions and camera settings might have affected the quality of the photos and thereby the esthetic scores.

Radiographic bone loss measurements come with a degree of imprecision, despite calibration and the use of dedicated imaging software. Follow‐up time at T1 ranged from 1 to 9.5 years between patients. Therefore, we cannot generalize about the results in the long term. The sample might not be representative of the broader population of patients suffering TDI, as the authors' department only treats patients already planned for implant placement because of early traumatic tooth loss.

Regarding the correlation analysis, multiple dependent variables were tested for effect on esthetic outcomes, necessitating the use of multiple different statistical tests. Because no Bonferroni correction was performed, the results are at risk of type I error and should therefore be interpreted with caution. Subjective esthetic evaluation was only done at follow‐up; therefore, no conclusion can be drawn on the effect of treatment on subjective satisfaction.

Despite the nature of the study design, the data are relatively homogenous and of high quality because of the standardized protocol used at baseline and during the follow‐up visits. Outcome assessment was strengthened using a validated esthetic index (Dueled et al. 2009). Keeping in mind the limitations, the overall results seem valid as indicators of which variables may affect the outcome.

In conclusion, the present study has indicated that multiple patient factors, biological factors, and timing of treatment affect the esthetic outcomes of implant treatment in the anterior maxilla after traumatic tooth loss. These outcomes are relevant because they strongly influence the success of the treatment, which should be defined by objective goals and patient satisfaction. Therefore, if esthetic outcomes can be better predicted, treatment planning and patient alignment of expectations may be improved.

Future research is encouraged to prospectively focus on the esthetic success of implant treatment in the anterior maxilla after traumatic tooth loss. Prospectively designed studies are needed to investigate the cause–effect relationships between variables and the esthetic outcomes and to determine their relative importance in treatment planning.

## Author Contributions

**Frej Nørgaard Petersen:** conceptualization and design, data collection, data analysis and interpretation, drafting manuscript, and agreement for accountability. **Morten Dahl:** conceptualization and design, data analysis and interpretation, critical revision of the manuscript, approval of the manuscript, and agreement for accountability. **Mandana Hosseini:** data analysis and interpretation, critical revision of the manuscript, approval of the manuscript, and agreement for accountability. **Simon Storgård Jensen:** conceptualization and design, data collection, data analysis and interpretation, critical revision of the manuscript, approval of the manuscript, and agreement for accountability.

## Conflicts of Interest

The authors declare no conflicts of interest.

## Supporting information

Appendix A.

## Data Availability

The data that support the findings of this study are available on request from the corresponding author. The data are not publicly available due to privacy or ethical restrictions. The data which this study is based upon may be requested from the corresponding author. Due to GDPR reasons the data will not be published.
