# Supplementary material for: Implant Treatment After Traumatic Tooth Loss: A Retrospective Cohort Study of Survival, Esthetic, and Patient‐Reported Outcome
Source: Clin Exp Dent Res. 2025 Nov 18;11(6):e70221. doi: 10.1002/cre2.70221 (PMC12626377; doi:10.1002/cre2.70221)
Supplement: Supplementary file 1 — Appendix A. [file CRE2-11-e70221-s001.docx]

**Appendix A – questionnaire used for assessment of patient-related outcome measure**

| **General questions** | Satisfied/never | Acceptable/Sometimes | Dissatisfied/often |  |
| --- | --- | --- | --- | --- |
| How satisfied are you in general with the aesthetics of your dental crown | 41 | 7 | 1 |  |
| How satisfied are you in general with the appearance of the gums around your implant and dental crown | 36 | 9 | 4 |  |
| Have you notices if one of your teeth looks different than the rest | 20 | 27 | 2 |  |
| Have you felt limited because of the appearance of your implant or crown | 41 | 6 | 2 |  |
| Have you ever avoided smiling because of the appearance of you r implant or crown | 44 | 2 | 3 |  |
| **Regarding the crown** |  |  |  |  |
| Are you satisfied with the colour of the crown? | 40 | 8 | 1 |  |
| If acceptable or dissatisfied, the reason being: | Too white | 5 | Too dark | 4 |
| Are you satisfied with the length of the crown? | 37 | 12 | 0 |  |
| If acceptable or dissatisfied, the reason being: | Too long | 2 | Too short | 9 |
| Are you satisfied with the width of the crown? | 46 | 2 | 1 |  |
| If acceptable or dissatisfied, the reason being: | Too narrow | 1 | Too wide | 1 |
| Are you satisfied with the position of the crown | 47 | 1 | 1 |  |
| If acceptable or dissatisfied, the reason being: | Placed too far inside the tooth arch | 0 | Placed too far outside of the tooth arch | 2 |
| **Regarding the gums** |  |  |  |  |
| Are you satisfied with the colour of the gums? | 44 | 3 | 2 |  |
| If acceptable or dissatisfied, the reason being: | Too dark | 4 | Too bright | 1 |
| Are you satisfied with the volume of the gums? | 39 | 7 | 3 |  |
| If acceptable or dissatisfied, the reason being: | Too flat | 8 | Too bulky | 1 |
| Are you satisfied with the contour of the gums? | 41 | 7 | 1 |  |
| If acceptable or dissatisfied, the reason being: | Too far up | 7 | Too far down | 0 |
| Are you satisfied with the fill of the gums between your teeth? | 37 | 9 | 3 |  |
| If acceptable or dissatisfied, the reason being: | Too much fill | 0 | Too little fill | 11 |
